# Effects of COVID-19 Lockdown on Movement Disorders Patients With Deep Brain Stimulation: A Multicenter Survey

**DOI:** 10.3389/fneur.2020.616550

**Published:** 2020-12-16

**Authors:** Carla Piano, Francesco Bove, Tommaso Tufo, Isabella Imbimbo, Danilo Genovese, Alessandro Stefani, Massimo Marano, Antonella Peppe, Livia Brusa, Rocco Cerroni, Francesco Motolese, Enrico Di Stasio, Marianna Mazza, Antonio Daniele, Alessandro Olivi, Paolo Calabresi, Anna Rita Bentivoglio, Lazio DBS Study Group

**Affiliations:** ^1^Neurology Unit, Fondazione Policlinico Universitario A. Gemelli IRCCS, Rome, Italy; ^2^Department of Neurosciences, Università Cattolica del Sacro Cuore, Rome, Italy; ^3^Neurosurgery Unit, Fondazione Policlinico Universitario A. Gemelli IRCCS, Rome, Italy; ^4^Fondazione Don Carlo Gnocchi Onlus, Milan, Italy; ^5^Department of System Medicine, Unità Operativa Semplice Dipartimentale (UOSD) Parkinson, University of Rome Tor Vergata, Rome, Italy; ^6^Neurology, Neurophysiology and Neurobiology Unit, Department of Medicine, Università Campus Bio-Medico di Roma, Rome, Italy; ^7^IRCCS Santa Lucia Foundation, Rome, Italy; ^8^Neurology Unit, S. Eugenio Hospital, Rome, Italy; ^9^Unit of Chemistry, Biochemistry and Clinical Molecular Biology, Fondazione Policlinico Universitario A. Gemelli IRCCS, Rome, Italy; ^10^Institute of Biochemistry and Clinical Biochemistry, Università Cattolica del Sacro Cuore, Rome, Italy; ^11^Department of Geriatrics, Neuroscience and Orthopedics, Institute of Psychiatry and Psychology, Fondazione Policlinico Universitario A. Gemelli IRCCS, Università Cattolica del Sacro Cuore, Rome, Italy

**Keywords:** COVID-19, SARS-CoV-2, deep brain stimulation, Parkinson's disease, dystonia

## Abstract

**Background:** The containment measures taken by Italian government authorities during the severe acute respiratory syndrome coronavirus 2 (SARS-CoV-2) pandemic caused the interruption of neurological activities of outpatient clinics. Vulnerable patients, as Parkinson's disease (PD) and dystonic patients with deep brain stimulation (DBS), may have an increased risk of chronic stress related to social restriction measures and may show a potential worsening of motor and psychiatric symptoms.

**Methods:** This cross-sectional multicenter study was carried out during the SARS-CoV-2 pandemic and was based on a structured survey administered during a telephone call. The questionnaire was designed to gather motor and/or psychiatric effects of the lockdown and coronavirus disease 2019 (COVID-19) epidemiologic information in PD and dystonic patients with a functioning DBS implant.

**Results:** One hundred four patients were included in the study, 90 affected by PD and 14 by dystonia. Forty-nine patients reported a subjective perception of worsening of global neurological symptoms (motor and/or psychiatric) related to the containment measures. In the multivariate analysis, having problems with the DBS device was the only independent predictor of motor worsening [odds ratio (OR) = 3.10 (1.22–7.91), *p* = 0.018]. Independent predictors of psychiatric worsening were instrumental activities of daily living (IADL) score [OR = 0.78 (0.64–0.95), *p* = 0.012] and problems with DBS [OR = 5.69 (1.95–16.62), *p* = 0.001]. Only one patient underwent nasopharyngeal swabs, both negative, and no patient received a diagnosis of COVID-19.

**Conclusions:** Lockdown restriction measures were associated with subjective worsening of motor and psychiatric symptoms in PD and dystonic patients treated with DBS, and they may have exacerbated the burden of neurological disease and increased the chronic stress related to the DBS management.

## Introduction

In early 2020, severe acute respiratory syndrome coronavirus 2 (SARS-CoV-2) infection has spread worldwide, becoming a pandemic. Coronavirus disease 2019 (COVID-19) commonly presents with fever, cough, and dyspnea; the most severe complication of the infection is the acute respiratory distress syndrome (1). Many government authorities took emergency containment measures to reduce viral transmission (2). In Italy, according to Prime Minister Decree of March 11, 2020, the containment measures recommended a reduction of routine hospital activities: activities of outpatient clinics were stopped, and admittance of patients affected by neurological disorders was allowed only for emergency conditions. Elderly individuals with preexisting comorbidities, including movement disorders, are fragile patients due to their higher risk of infections and poor outcome of disease management (3). Deep brain stimulation (DBS) is a well-established second-level treatment for patients with severe Parkinson's disease (PD) and dystonia who show poor response to pharmacological treatment (4). These patients are routinely monitored with close follow-up, given the need for periodic adjustments of stimulation parameters to optimize motor control and for periodic checks of implantable pulse generator (IPG) functionality and battery status in order to reduce the risk of device-related complications (5, 6). In addition, PD and dystonic patients treated with DBS often need a close follow-up of possible psychiatric symptoms (depression, anxiety, apathy, impulse control disorders, psychosis, disorders of sleep, and wakefulness) (7). For the aforementioned reasons, patients with movement disorders are exposed to an increased risk of chronic stress related to social restriction measures and may show a potential worsening of motor and psychiatric symptoms (8–10). Aim of the present study was to analyze the effects of the lockdown on this vulnerable category of patients affected by PD and dystonia treated with DBS and, secondarily, to investigate the prevalence of COVID-19 suggestive symptoms in this population.

## Patients and Methods

This cross-sectional multicenter study was carried out during the SARS-CoV-2 pandemic and was promoted by the Neurology Clinic of the Fondazione Policlinico Universitario A. Gemelli IRCCS in Rome (Italy), in collaboration with other neurology clinics based in Lazio region with good expertise on the management of DBS for movement disorders.

### Study Population

#### Inclusion Criteria

PD and dystonic patients who received a DBS implant, which was functioning during the COVID-19 lockdown, and followed in the DBS centers of Lazio region were asked to participate in a telephone survey. Patients were included regardless of the DBS target, the hospital, and the year in which implantation of the neurostimulator was carried out.

#### Exclusion Criteria

Patients were excluded if they were unable to provide informed and valid consent at the time of the interview, if they show cognitive decline, and if they did not speak Italian fluently.

### Survey Design and Testing

The study was based on a structured survey administered during a telephone call carried out as part of the close follow-up scheduled for the included patients. Surveys started on April 28, 2020, and ended on May 12, 2020. A questionnaire was employed, aimed at gathering the following data:

- demographic and clinical data related to PD and dystonia (age, sex, age at disease onset, disease duration, presence of psychiatric symptoms prior to the lockdown, preexisting hyposmia, PD phenotype, current treatment of the neurological disorder)
- information about DBS (target, years from the DBS surgery)
- assumption of medications potentially interfering with SARS-CoV-2 infection
- concomitant diseases and disability measures [activities of daily living (ADL) and instrumental activities of daily living (IADL) scales (11, 12), Hoehn and Yahr (H&Y) scale for PD patients (13), Clinical Global Impression (CGI) Severity scale (14)]
- COVID-19-related questions [including a history of COVID-19-suggestive symptoms in the last 4 months (fever, cough, dyspnea, diarrhea, abdominal pain, pharyngodynia, worsening of hyposmia, hearing loss) and additional information related to SARS-CoV-2 pandemic (testing by nasopharyngeal swabs, COVID-19 diagnosis, contact with COVID-19+ subjects, presence in endemic areas for COVID-19, presence in residential care home, respect of social restriction measures according to Prime Minister Decree of March 11, 2020, flu vaccination)]
- effects of the lockdown (all of the following were yes/no answer questions) [discontinuation of outpatient neurological visits or physiotherapy (motor physical therapy), difficulties in finding medications, need of urgent outpatient neurological visit, difficulties in the management of the DBS device (rechargeable systems and/or patients with handheld controller to turn the DBS system on and off), feeling of insecurity about the DBS device, worsening of the relationship with own body, subjective perception of worsening of global neurological symptoms (motor and/or psychiatric), sleep disorders, depression, panic attacks, persecutory delusions, visual hallucinations, suicidal ideation, impulsive–compulsive behaviors, increased consumption of coffee and tea]
- changes on CGI Improvement scale (14) related to the lockdown period

For patients who complained one or both “difficulties in the management of the DBS device” and “feeling of insecurity about the DBS device,” the cumulative variable “problems with the DBS device” was considered.

The variables related to the effects of the lockdown were assessed to detect emergent symptoms and/or changes of preexisting symptoms after March 11, 2020.

### Statistical Analysis

All statistical analyses were carried out using the Statistical Package for Social Science (SPSS) program, version 25.0 (IBM Co., Armonk, NY). Data were analyzed for normality of distribution using the Kolmogorov–Smirnov test of normality and were expressed as mean [±standard deviation (SD)] for continuous variables or as frequencies (*n*, %) for categorical variables according to a neurological diagnosis. The Mann–Whitney rank sum test was used to compare continuous variables between diagnostic subgroups (PD and dystonia) and between patients with or without subjective perception of worsening of neurological symptoms. The χ^2^ test or Fisher's exact test was used for categorical variables. Phi coefficients were calculated to estimate correlation strengths between motor/psychiatric worsening and the other binomial variables evaluating the effects of the lockdown. Finally, multivariate binary logistic analysis was performed to evaluate the relationship between the worsening of motor or psychiatric symptoms and clinical findings. The coefficients obtained from the logistic regression were expressed in terms of odds ratio (OR) with 95% confidence intervals. All statistical tests were two-tailed; statistical significance was defined as *p* value < 0.05, and the effect size was also reported.

### Standard Protocol Approvals, Registration, and Patient Consent

The study and the survey questions were reviewed and approved by local ethical committee. Because of the biological risks related to the pandemic, after receiving a copy of written informed consent by mail or fax, patients were asked to carefully read, sign, and send it by mail or fax to the referral hospital.

### Data Availability

Anonymized data will be shared with qualified external researchers after approval of their requests.

## Results

One hundred four patients treated with DBS were asked to participate in the survey. Since all agreed to participate, met the inclusion criteria, and no one had exclusion criteria, all 104 patients were included in the study. Ninety patients were affected by PD and 14 patients by dystonia (including 10 patients with idiopathic dystonia and four patients with secondary dystonia). In the total sample, the male/female ratio was 64/40, the mean age was 61 years (SD ±12), and the mean disease duration was 19 years (SD ±8). All dystonic patients underwent globus pallidus internus (GPi) DBS, while the DBS target was different among PD patients: 85 patients were implanted in the subthalamic nucleus (STN), three patients in the GPi, and two patients in the thalamic ventral intermediate nucleus (Vim).

For each diagnostic group, demographic and disease clinical data, disability scales, concomitant medical conditions, and neurological medications are reported in Table 1. When the subgroups of PD and dystonic patients were compared for demographic and disease clinical features, significant differences were found for age (62 ± 10 vs. 53 ± 16 years, *p* = 0.038, effect size = 0.82–large), age at disease onset (44 ± 9 vs. 29 ± 19 years, *p* = 0.007, effect size = 1.39–large), disease duration (18 ± 7 vs. 24 ± 10 years, *p* = 0.020, effect size = 0.81–large), scores on ADL (4.8 ± 1.8 vs. 5.9 ± 0.5, *p* = 0.012, effect size = 0.65–medium), IADL (4.4 ± 3.0 vs. 7.1 ± 2.1, *p* = 0.001, effect size = 0.93–large), and CGI Severity scale (4.0 ± 1.3 vs. 2.9 ± 1.6, *p* = 0.016, effect size = 0.82–large).

**Table 1:** Demographic and disease clinical data, disability scales, concomitant medical conditions, and specific neurological medications for each diagnostic group.

	**PD**	**Dystonia**	**Total**
	**(*n* = 90)**	**(*n* = 14)**	**(*n* = 104)**
**Demographic features**			
Age (years)	62 ± 10	53 ± 16	61 ± 12
Male, *n* (%)	58 (64%)	6 (43%)	64 (62%)
Age at disease onset (years)	44 ± 9	29 ± 19	42 ± 12
Disease duration (years)	18 ± 7	24 ± 10	19 ± 8
**Information about DBS**			
DBS target (STN/GPi/Vim), *n* (%)	85/3/2	0/14/0	85/17/2
	(95%/3%/2%)	(0%/100%/0%)	(82%/16%/2%)
Years from DBS surgery	7 ± 6	9 ± 7	7 ± 6
**Disease clinical features**			
Psychiatric symptoms, *n* (%)	58 (64%)	6 (43%)	64 (62%)
Hyposmia, *n* (%)	66 (73%)	1 (7%)	67 (64%)
Rigid-akinetic phenotype^*^, *n* (%)	46 (52%)	–	–
Tremor-dominant phenotype^*^,	23 (26%)	–	–
*n* (%)			
Mixed phenotype^*^, *n* (%)	20 (22%)	–	–
**Disability scales**			
ADL	4.8 ± 1.8	5.9 ± 0.5	4.9 ± 1.7
IADL	4.4 ± 3.0	7.1 ± 2.1	4.8 ± 3.0
H&Y stage	3.1 ± 0.7	–	–
CGI Severity scale	4.0 ± 1.3	2.9 ± 1.6	3.9 ± 1.4
**Concomitant medical conditions**			
Hypertension, *n* (%)	18 (20%)	2 (14%)	20 (19%)
Heart diseases, *n* (%)	8 (9%)	0	8 (8%)
COPD, *n* (%)	5 (6%)	0	5 (5%)
Diabetes, *n* (%)	2 (2%)	1 (7%)	3 (3%)
Cancer, *n* (%)	6 (7%)	0	6 (6%)
Chronic kidney disease, *n* (%)	2 (2%)	0	2 (2%)
Obesity, *n* (%)	13 (14%)	1 (7%)	14 (13%)
Smoke, *n* (%)	30 (35%)	5 (38%)	35 (34%)
**Neurological medications**			
Levodopa, *n* (%)	84 (93%)	0	84 (78%)
MAOIs, *n* (%)	64 (71%)	0	64 (59%)
COMTIs, *n* (%)	36 (40%)	0	36 (35%)
Dopamine agonists, *n* (%)	42 (47%)	0	42 (40%)
Amantadine, *n* (%)	18 (20%)	0	18 (17%)
Anticholinergics, *n* (%)	4 (4%)	3 (21%)	7 (7%)
BoNT *n* (%)	1 (1%)	2 (14%)	3 (3%)
Benzodiazepines, *n* (%)	27 (30%)	5 (36%)	32 (31%)
Antidepressants, *n* (%)	33 (37%)	5 (36%)	38 (37%)
Antipsychotics, *n* (%)	30 (33%)	2 (14%)	32 (31%)
Mood stabilizers, *n* (%)	15 (17%)	3 (21%)	18 (17%)

* *The classification in the three different phenotypes of PD (rigid-akinetic, tremor-dominant, and mixed) was made according the tremor/akinetic-rigid (T/AR) ratio (15)*.

*PD, Parkinson's disease; DBS, deep brain stimulation; STN, subthalamic nucleus; GPi, globus pallidus internus; Vim, ventral intermediate nucleus; ADL, activities of daily living; IADL, instrumental activities of daily living; H&Y, Hoehn and Yahr; COPD, chronic obstructive pulmonary disease; MAOIs, monoamine oxidase inhibitors; COMTIs, catechol-O-methyltransferase inhibitors; BoNT, botulinum neurotoxin*.

### Coronavirus Disease 2019-Related Questions

Distribution of symptoms suggestive of COVID-19 in the last 4 months, epidemiologic data, and medications potentially interfering with the SARS-CoV-2 infection are reported in Table 2. Twenty-six patients reported one or more symptoms suggestive of COVID-19, while only five of them referred three or more of these symptoms. The most frequent symptom was cough, reported by 17 patients. Only one patient underwent (twice) nasopharyngeal swabs, both negative, and no patient received a diagnosis of COVID-19. In our sample, there was no correlation between symptoms suggestive of SARS-CoV-2 infection and assumption of angiotensin-converting enzyme (ACE) inhibitors, nonsteroidal anti-inflammatory drugs (NSAIDs), or immunosuppressant therapy.

**Table 2:** Symptoms suggestive of COVID-19 in the last 4 months, epidemiologic data, and medications potentially interfering with SARS-CoV-2 infection for each diagnostic group.

	**PD (*n* = 90)**	**Dystonia (*n* = 14)**	**Total (*n* = 104)**
**Symptoms suggestive of COVID-19**			
Fever, *n* (%)	5 (6%)	1 (7%)	6 (6%)
Cough *n* (%)	15 (17%)	2 (14%)	17 (16%)
Dyspnea, *n* (%)	3 (3%)	0	3 (3%)
Diarrhea, *n* (%)	5 (6%)	0	5 (5%)
Abdominal pain, *n* (%)	5 (6%)	0	5 (5%)
Pharyngodynia, *n* (%)	5 (6%)	1 (7%)	6 (6%)
Hyposmia worsening, *n* (%)	2 (2%)	0	2 (2%)
Hearing loss, *n* (%)	2 (2%)	0	2 (2%)
**Additional information related to SARS-CoV-2 pandemic**			
Testing by nasopharyngeal swabs	1 (1%)	0	1 (1%)
COVID-19 diagnosis	0	0	0
Contact with COVID-19+ subjects	3 (3%)	0	3 (3%)
Presence in endemic areas for COVID-19, *n* (%)	2 (2%)	0	2 (2%)
Presence in residential care home, *n* (%)	1 (1%)	0	1 (1%)
Respect of social restriction measures, *n* (%)	84 (93%)	13 (93%)	97 (93%)
Flu vaccination, *n* (%)	26 (29%)	2 (14%)	28 (27%)
**Medications potentially interfering with SARS-CoV-2 infection**			
ACE inhibitors, *n* (%)	2 (2%)	2 (14%)	4 (4%)
NSAIDs, *n* (%)	3 (4%)	0	3 (3%)
Immunosuppressants, *n* (%)	3 (4%)	0	3 (3%)

*PD, Parkinson's disease; NSAIDs, nonsteroidal anti-inflammatory drugs; ACE, angiotensin-converting enzyme; COVID-19, coronavirus disease 2019; SARS-CoV-2, severe acute respiratory syndrome coronavirus 2*.

No significant differences were found between the two diagnostic subgroups (PD and dystonia) as to COVID-19-suggestive symptoms or additional information related to SARS-CoV-2 pandemic.

### Effects of the Lockdown on Disease Burden

Information related to the impact of the lockdown on disease burden is reported in Table 3. Forty-nine patients out of 104 (47%) reported a subjective perception of worsening of global neurological symptoms related to the containment measures: 20 patients (19%) reported a worsening of motor symptoms; five patients (5%), a worsening of psychiatric symptoms; 24 patients (23%), a worsening of both motor and psychiatric symptoms. The overall worsening of the health status was also confirmed by the mean score on the CGI Improvement scale (4.6 ± 1.3). Ninety-two patients (88%) lost the scheduled follow-up visit, 60 (58%) discontinued physiotherapy, and 18 (17%) needed urgent outpatient neurological visit. Eighteen patients (17%) reported difficulties in the management of the DBS device, 26 (25%) reported feeling of insecurity about the DBS device, while 28 (27%) reported a worsening in the relationship with their own body as compared to the period immediately preceding the lockdown. Thirty-four patients (33%) complained of sleep disorders, 39 (37%) depression, 13 (12%) panic attacks, nine (9%) persecutory delusions, 13 (12%) visual hallucinations, and eight (8%) suicidal ideation. Moreover, 21 patients (20%) reported impulsive–compulsive behaviors as increase of shopping online, video game playing, and punding. Thirty-five patients (34%) reported an increased coffee and tea consumption. Among the 21 patients who complained worsening of impulsive–compulsive behaviors, 10 were on treatment with dopamine agonists. The proportion of patients on treatment with dopamine agonists was not significantly higher in the subgroup with worsening of impulsive–compulsive behaviors than in the other subgroup of patients (48 vs. 39%, respectively, *p* = 0.6).

**Table 3:** Effects of the lockdown on disease burden for each diagnostic group.

	**PD (*n* = 90)**	**Dystonia (*n* = 14)**	**Total (*n* = 104)**
**Effects of the lockdown**			
Perception of global neurological worsening, *n* (%)	44 (49%)	5 (36%)	49 (47%)
Perception of motor worsening, *n* (%)	39 (43%)	5 (36%)	44 (42%)
Perception of psychiatric worsening, *n* (%)	25 (28%)	4 (29%)	29 (28%)
Discontinuation of outpatient neurological visits, *n* (%)	78 (87%)	14 (100%)	92 (88%)
Discontinuation of physiotherapy, *n* (%)	55 (61%)	5 (36%)	60 (58%)
Need for urgent outpatient neurological visit, *n* (%)	16 (18%)	2 (14%)	18 (17%)
Difficulties in finding medications, *n* (%)	3 (3%)	0	3 (3%)
Difficulties in the management of the DBS device, *n* (%)	13 (14%)	5 (36%)	18 (17%)
Feeling of insecurity about the DBS device, *n* (%)	21 (23%)	5 (36%)	26 (25%)
Problems with the DBS device, *n* (%)	27 (30%)	7 (50%)	34 (33%)
Worsening of the relationship with own body, *n* (%)	24 (27%)	4 (29%)	28 (27%)
Sleep disorders, *n* (%)	29 (32%)	5 (36%)	34 (33%)
Mood impairment, *n* (%)	38 (42%)	7 (50%)	45 (43%)
Depression, *n* (%)	37 (41%)	2 (14%)	39 (37%)
Panic attacks, *n* (%)	11 (12%)	2 (14%)	13 (12%)
Persecutory delusions, *n* (%)	7 (8%)	2 (14%)	9 (9%)
Visual hallucinations, *n* (%)	12 (13%)	1 (7%)	13 (12%)
Suicidal ideation, *n* (%)	7 (8%)	1 (7%)	8 (8%)
Impulsive–compulsive behaviors, *n* (%)	18 (20%)	3 (21%)	21 (20%)
Shopping online, *n* (%)	4 (4%)	1 (7%)	5 (5%)
Video game playing, *n* (%)	8 (9%)	3 (21%)	11 (11%)
Punding, *n* (%)	8 (9%)	0	8 (8%)
Increased consumption of coffee and tea, *n* (%)	31 (34%)	4 (29%)	35 (34%)
CGI Improvement scale	4.6 ± 1.1	4.2 ± 1.1	4.6 ± 1.3

*PD, Parkinson's disease; DBS, deep brain stimulation; CGI, Clinical Global Impression*.

No significant differences were found between the two diagnostic subgroups (PD vs. dystonic patients) as to the effects of the lockdown.

Considering the subgroup of 26 patients reporting one or more symptoms suggestive of COVID-19 and comparing this one with the subgroup of patients without symptoms suggestive of COVID-19, no significant differences were found in worsening of motor or psychiatric symptoms.

Significant correlations were found between motor worsening and discontinuation of physiotherapy (phi coefficient = 0.22, *p* = 0.024, effect size = 0.45–small) and between worsening of psychiatric symptoms and chronic benzodiazepine intake (phi coefficient = 0.28, *p* = 0.004, effect size = 0.59–medium). Significant correlations were also observed between both motor and psychiatric worsening and need for urgent outpatient neurological visit (phi coefficient = 0.38, *p* < 0.001, effect size = 1.06–large and phi coefficient = 0.40, *p* < 0.001, effect size = 0.99–large, respectively), difficulties in the management of the DBS device (phi coefficient = 0.23, *p* = 0.021, effect size = 0.60–medium and phi coefficient = 0.23, *p* = 0.021, effect size = 0.60–medium, respectively), feeling of insecurity about the DBS device (phi coefficient = 0.27, *p* = 0.006, effect size = 0.63–medium and phi coefficient = 0.28, *p* = 0.004, effect size = 0.63–medium, respectively), worsening of the relationship with own body (phi coefficient = 0.45, *p* < 0.001, effect size = 1.04–large and phi coefficient = 0.40, *p* < 0.001, effect size = 0.86–large, respectively), sleep disorders (phi coefficient = 0.27, *p* = 0.005, effect size = 0.59–medium and phi coefficient = 0.44, *p* < 0.001, effect size = 0.91–large, respectively), depression (phi coefficient = 0.22, *p* = 0.024, effect size = 0.46–small and phi coefficient = 0.45, *p* < 0.001, effect size = 0.99–large, respectively).

No difference was found on disability scales between the subgroups of patients with and without worsening of motor symptoms. By contrast, the subgroup with worsening of psychiatric symptoms, as compared to the subgroup without psychiatric worsening, presented significantly lower scores on IADL (3.7 ± 3.0 vs. 5.2 ± 2.9, *p* = 0.0436, effect size = 0.50–small) and higher scores on CGI Severity scale (4.5 ± 1.0 vs. 3.7 ± 1.4, *p* = 0.0081, effect size = 0.71–medium).

In the reduced models of multivariate logistic regression analysis, having problems with the DBS device (difficulties in the management of the DBS device or feeling of insecurity about the DBS device) was the only independent predictor of motor worsening [OR = 3.10 (1.22–7.91), *p* = 0.018]. Independent predictors of psychiatric worsening were IADL score [OR = 0.78 (0.64–0.95), *p* = 0.012] and problems with DBS [OR = 5.69 (1.95–16.62), *p* = 0.001]. Other variables included in the models were age, sex, diagnosis (PD or dystonia), and disease duration (Table 4).

**Table 4:** Multivariate logistic regression models of factors predicting motor and psychiatric worsening during the lockdown.

**Variable**	**OR**	**95% CI (lower–upper)**	***p* value**
**Motor worsening**			
Age	0.99	0.95–1.03	0.6
Male sex	1.51	0.60–3.77	0.4
PD diagnosis	1.40	0.31–6.23	0.7
Disease duration	0.98	0.92–1.04	0.5
IADL	0.95	0.81–1.11	0.5
Problems with DBS	3.10	1.22–7.91	0.018^*^
**Psychiatric worsening**			
Age	0.98	0.94–1.03	0.4
Male sex	0.71	0.26–1.95	0.5
PD diagnosis	0.86	0.15–4.97	0.9
Disease duration	1.00	0.93–1.07	0.9
IADL	0.78	0.64–0.95	0.012^*^
Problems with DBS	5.69	1.95–16.62	0.001^*^

* *p < 0.05*.

*OR, odds ratio; CI, confidence interval; PD, Parkinson's disease; DBS, deep brain stimulation; IADL, instrumental activities daily living*.

## Discussion

The study, based on a telephone survey administered by neurologists of the DBS movement disorders network in Lazio region, attempted to investigate the consequences of the COVID-19 lockdown on the perception of neurological status (motor and psychiatric symptoms) in patients affected by PD and dystonia treated with DBS.

The demographic and clinical characteristics between the two diagnostic subgroups differed significantly: as compared to PD patients, dystonic patients showed an earlier age at onset, a longer disease duration, and a milder disability.

Although patients with neurological disorders, especially in late disease stages, might be particularly at risk for COVID-19 complications (16–18), in our sample, only ¼ of patients reported symptoms suggestive of SARS-CoV-2 infection. One patient underwent nasopharyngeal swabs, and none received a diagnosis of COVID-19. No patient with symptoms suggestive of COVID-19 needed specific treatments, hospitalization, or respiratory assistance.

Half of the patients in our sample reported a subjective perception of global neurological worsening, which required urgent outpatient neurological visit in 18 cases. A mean CGI Improvement score >4 after the lockdown period confirmed these data. In most patients, worsening of health status seemed to result from discontinuation of neurological visits and physiotherapy, as reported for patients with chronic neurological diseases (19, 20). A significant number of patients presented a worsening of sleep quality, mood, and behavioral disturbances, including onset or worsening of impulsive–compulsive behaviors. As well as worsening of impulsive–compulsive behaviors was not related to treatment with dopamine agonists, it may be a consequence of restriction measures more than an adverse effect induced by dopamine agonists. Indeed, 13 patients reported visual hallucinations during the weeks of social isolation probably induced by a home “hospitalization” phenomenon (21) and exacerbated by sleep disturbances (22).

No significant differences were found between the two diagnostic subgroups (PD vs. dystonic patients) as to the effects of the lockdown. Furthermore, in the multivariate analysis, the specific neurological disease was not an independent predictor of motor or psychiatric worsening during the lockdown.

No significant differences were found in worsening of motor or psychiatric symptoms comparing the subgroup of patients with and without symptoms suggestive of COVID-19.

Significant correlations were found between the subjective perception of motor worsening and discontinuation of physiotherapy treatment, although with a small effect size, and between worsening of psychiatric symptoms and chronic intake of benzodiazepines, with a medium effect size. These findings point out the beneficial effects of physiotherapy in patients with movement disorders and the importance of strategies focused on remote rehabilitation treatment (23). Indeed, a closer psychiatric follow-up would be recommended for patients with chronic use of benzodiazepines, who have an increased risk of developing depressive symptoms (24).

In our study, problems with the DBS device (difficulties in the management of the DBS device and feeling of insecurity about the DBS device) were independent predictors of motor and psychiatric worsening. Such difficulties may be chronic stressful factors that induce depression, anxiety, and insomnia (25). Therefore, DBS played an important role in impairing motor and psychiatric symptoms during the lockdown, independently from the underlying neurological disease. Greater disability (lower IADL score) due to the chronic neurological disease was another independent predictor of psychiatric worsening. On the other hand, demographic factors, disease duration, and neurological diagnosis (PD or dystonia) did not contribute to the global worsening during the lockdown.

These findings suggest that the effects of social restriction measures seriously impact on patients with chronic neurological disease and, in particular, in carriers of a DBS stimulator with greater disability. They also highlight the importance of management programs in the post-epidemic phase with the implementation of telemedicine, remote rehabilitation treatment, and technologies for remote DBS monitoring and programming (9, 26, 27).

### Limitations

This study has several limits. First, since data collection was carried out by means of telephone contacts and not by a face-to-face assessment, the results of the survey might have been influenced by uncontrolled biases. Second, the effects of the lockdown were only assessed by subjective perception and not by validated scales for motor and psychiatric symptoms because of the lack of specific evaluation just before the lockdown. Furthermore, subjective perception of motor and/or psychiatric worsening may be influenced by depressive mood, which had a high prevalence in our sample and is typical of the pandemic situation. In fact, there is a positive correlation between depression and subjective perception of motor and/or psychiatric worsening, thus the patients might have overestimated the real worsening during the pandemic. Another limitation of the study is the small sample size for dystonic patients. Finally, although the multivariate analysis showed that DBS played an independent role in motor and psychiatric worsening, future comparative studies between carriers and noncarriers of DBS should be made.

## Conclusions

Our findings suggest that lockdown restriction measures were associated with subjective worsening of motor and psychiatric symptoms in PD and dystonic patients treated with DBS, and that such measures may have exacerbated the burden of neurological disease and increased the chronic stress related to the DBS management.

### Lazio DBS Study Group

Maria Concetta Altavista, Neurology Unit, San Filippo Neri Hospital ASL Roma 1, Roma, Italy

Marco Ciavarro, IRCCS Neuromed, Pozzilli, Italy

Francesca Cortese, Neurology Unit, San Filippo Neri Hospital ASL Roma 1, Roma, Italy

Manuela D'Ercole, Neurosurgery, Fondazione Policlinico Universitario A. Gemelli IRCCS, Rome, Italy

Lazzaro Di Biase, Unit of Neurology, Neurophysiology and Neurobiology, Department of Medicine, Campus Bio-Medico of Rome University, Rome, Italy

Maria Francesca De Pandis, Institute for Research and Medical Care IRCCS San Raffaele Cassino, Cassino, Italy

Daniela Di Giuda, Nuclear Medicine, Fondazione Policlinico Universitario A. Gemelli IRCCS, Università Cattolica del Sacro Cuore, Rome, Italy

Giovanni Fabbrini, Department of Human Neurosciences, Sapienza University of Rome, Italy; IRCCS Neuromed Institute, Pozzilli, Italy

Alessandro Izzo, Neurosurgery, Fondazione Policlinico Universitario A. Gemelli IRCCS, Rome, Italy

Rosa Liperoti, Geriatrics, Fondazione Policlinico Universitario A. Gemelli IRCCS, Rome, Italy

Giuseppe Marano, Institute of Psychiatry and Psychology, Department of Geriatrics, Neuroscience and Orthopedics, Fondazione Policlinico Universitario A. Gemelli IRCCS, Università Cattolica del Sacro Cuore, Rome, Italy

Nicola Modugno, IRCCS Neuromed, Pozzilli, Italy

Michela Orsini, Clinical Psychology, Fondazione Policlinico Universitario A. Gemelli IRCCS, Rome, Italy

Michele Paradiso, Department of Neurology, St John the Baptist Hospital, ACISMOM, Rome, Italy

Mariangela Pierantozzi, Neurology Unit, University Hospital “Tor Vergata”, Rome, Italy; Department of Systems Medicine, University of Rome Tor Vergata, Rome, Italy

Camilla Rocchi, Department of Systems Medicine, University of Rome Tor Vergata, Rome, Italy

Antonio Suppa, Department of Human Neurosciences, Sapienza University of Rome, Italy; IRCCS Neuromed Institute, Pozzilli, Italy

Laura Vacca, University and Institute for Research and Medical Care IRCCS San Raffaele, Rome, Italy

Rita Vadalà, NeuroRadiology, IRCCS Fondazione S Lucia, Rome, Italy

Michela Orsini, Neurology, Fondazione Policlinico Universitario A. Gemelli IRCCS, Rome, Italy

Fabio Viselli, Department of Neurology, St John the Baptist Hospital, ACISMOM, Rome, Italy.

## Data Availability Statement

The original contributions generated in the study are included in the article/supplementary materials, further inquiries can be directed to the corresponding author/s.

## Ethics Statement

The studies involving human participants were reviewed and approved by Fondazione Policlinico Universitario A. Gemelli IRCCS ethics committee. The patients/participants provided their written informed consent to participate in this study.

## Author Contributions

CP, FB, and ARB designed and conceptualized the study, interpreted the data, and drafted the manuscript. EDS analyzed the data and revised the manuscript for intellectual content. TT, II, DG, AS, MMar, AP, LB, RC, FM, MMaz, AD, AO, PC, and Lazio DBS Study Group collected the data and revised the manuscript for important intellectual content. All authors contributed to the article and approved the submitted version.

## Conflict of Interest

The authors declare that the research was conducted in the absence of any commercial or financial relationships that could be construed as a potential conflict of interest.
